# Generation and characterization of regulatory dendritic cells derived from murine induced pluripotent stem cells

**DOI:** 10.1038/srep03979

**Published:** 2014-02-05

**Authors:** Qi Zhang, Masayuki Fujino, Shizue Iwasaki, Hiroshi Hirano, Songjie Cai, Yuya Kitajima, Jinhua Xu, Xiao-Kang Li

**Affiliations:** 1 Department of Dermatology, Huashan hospital, Fudan University, Shanghai, China; 2 Division of Transplantation Immunology, National Research Institute for Child Health and Development, Tokyo, Japan; 3 AIDS Research Center, National Institute of Infectious Diseases, Tokyo, Japan; 4 Hasumi International Research Foundation, Tokyo, Japan

## Abstract

Regulatory dendritic cells (DCregs) represent a potential therapeutic tool for assessing a variety of immune overreaction conditions; however, current approaches for generating DCregs for therapeutic purposes are limited. We attempted to generate and characterize DCregs from murine induced pluripotent stem (iPS) cells. The iPS cells co-cultured with OP9 cells displayed mesodermally differentiated flat colonies. GM-CSF drove most of the colonies exhibiting a differentiated morphology. Thereafter, cells became morphologically heterologous under the effects of TGF-β and IL-10. Most of the floating cells developed an irregular shape with areas of protrusion. The generated iPS-DCregs demonstrated high CD11b/c and low CD40, CD80, CD86 and MHC-II expressions with a high antigen uptake ability and poor T-cell stimulatory function. Importantly, iPS-DCregs showed immune responsiveness regulation effects both *in vitro* and *in vivo* and the ability to generate regulatory T-cells *in vitro*. Our result illustrates a feasible approach for generating functional DCregs from murine iPS cells.

Dendritic cells (DCs) are unique professional major antigen presenting cells (APCs) capable of stimulating naive T cells in the primary immune response and are more potent APCs than monocytes/macrophages or B cells^1^. In addition, there is increasing evidence that DCs induce antigen-specific unresponsiveness or tolerance in central and periphery lymphoid organs *in situ*^2^. Due to their particular immune regulatory effects, separating them from conventional DCs (DCcons), these DCs are defined as tolerant DCs or regulatory DCs (DCregs)^3^. The main characteristics of DCregs include a weak expression of major histocompatibility complex (MHC) and co-stimulatory molecules and a weak ability to activate effector T cells, although they are able to generate and induce the proliferation of regulatory T cells (Tregs) and the anergy of autoreactive T cells to induce immune tolerance^2^,^3^,^4^. Therefore, DCregs have been tested for the treatment of posttransplant graft versus host disease (GvHD)^5^ and various autoimmune diseases^6^. Many trials of DC-based immunotherapy have shown this method to be reliable, safe and therapeutically efficient^7^. However, the major problem that prevents the routine clinical use of DCs in therapy for various diseases is the lack of plentiful and stable therapeutic DCs; therefore, it is essential to devise new methods for producing DCs in higher quantities and of greater purity^8^.

Several studies have reported methods of generating DCregs from murine bone morrow (BM) stem cells and human blood mononuclear cells. The DCregs generated using these methods display different phenotypes and characteristics. For instance, Sato et al. reported a method of generating DCregs from BM cells using treatment with granulocyte macrophage colony stimulating factor (GM-CSF) combined with interleukin (IL)-10 and transforming growth factor (TGF)-β. The DCregs produced using this method were able to generate Tregs and exhibited an immune regulation effect to ease GvHD reactions^9^. Farias et al. found that Vitamin D3 induces indoleamine 2,3-dioxygenase (IDO)-positive tolerogenic DCs from BM cells that enhance Tregs and reduce the severity of experimental autoimmune encephalomyelitis (EAE)^10^. Hong et al. found that constructed adenoviral vector coding suppressor of cytokine signaling (SOCS)-1 can efficiently increase the SOCS-1 gene expression in BM-DCcons and induce DCreg generation, possessing the therapeutic potential to prevent rejection in patients undergoing organ transplantation^11^. Summarizing these reports, the *in vitro* generation of DCregs has been achieved using many manipulations, such as the following three methods: 1) physiological mediators, including anti-inflammatory cytokines, such as IL-10, TGF-β1 and vascular endothelial growth factor (VEGF)^12^,^13^,^14^; 2) pharmacological agents, including anti-inflammatory agents (such as aspirin), cyclic adenosine monophosphate (cAMP) inducers (prostaglandin E2, histamine, β2 agonists, neuropeptides [calcitonin-gene-related peptide and vasoactive intestinal peptide]), vitamin D3 and immunosuppressive drugs (corticosteroids, cyclosporin A, rapamycin, deoxyspergualin and mycophenolate mofetil (MMF))^15^,^16^,^17^,^18^; and 3) genetic engineering of molecules, such as co-stimulatory molecules and cytokines, that can be transferred through viral or nonviral delivery systems or manipulated by selective gene silencing [antisense oligodeoxynucleotides (ODNs) and small interfering RNAs (siRNAs)]^19^,^20^,^21^.

In the present study, we describe a novel approach for generating a high number of functional DCregs from induced pluripotent stem (iPS) cells for downregulating the immune response. iPS cells were first reported by Takahashi and Yamanaka in 2006 and can be produced from various types of somatic cells via reprogramming by Yamanaka factors (Oct4, Sox2, Klf4 and c-Myc)^22^. iPS cells are very similar to embryonic stem (ES) cells in many respects, including gene expression patterns and pluripotent characteristics; however, they are not restricted by the same ethical concerns as ES cells. Therefore, iPS cells have great potential as a major cell source for producing various types of cells or organs in regenerative medicine^23^,^24^. The generation of DCcons from iPS cells has been reported by Senju et al., who succeeded in establishing an original method for generating DCcons from murine iPS cells with the aid of OP9 stromal cells and exogenous GM-CSF^25^. However, there have been no studies reporting the generation of DCregs from iPS cells. Therefore, we investigated the use of OP9 stromal cells as feeder cells accompanying the combination of exogenous GM-CSF and the anti-inflammatory cytokines IL-10 and TGF-β as culture conditions to generate DCregs from murine iPS cells (iPS-DCreg) and characterized the cells using morphological, related gene-expression and functional analyses.

## Results

### Generation of regulatory dendritic cells from iPS cells

**Figure 1:**
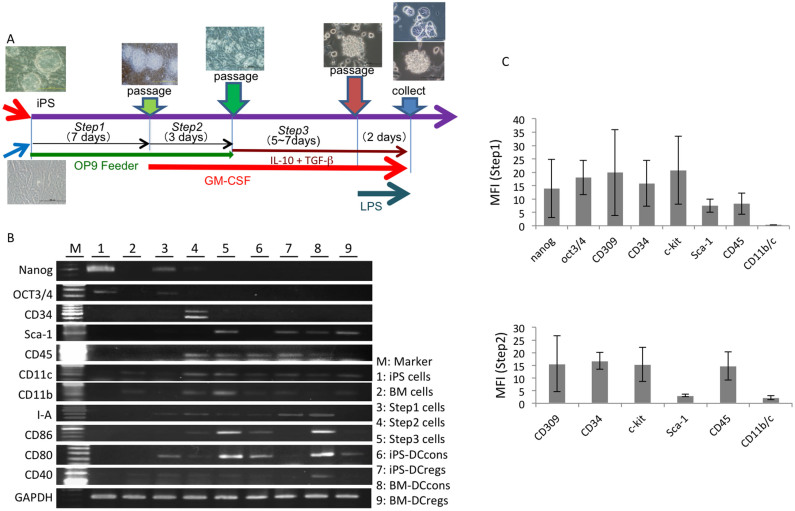
*Culture protocol and characteristics of regulatory dendritic cells generated from iPS cells.* (A) From left to right, this schematic model shows the culture protocol for each step of iPS-DC generation, with images of the cells under a microscope. The exogenous cytokines in the culture media are also shown. (B) The mRNA expression of the iPS cells in each step of differentiation into DCregs. The mRNA expressions of major molecules, including Nanog, OCT 3/4, CD34, Sca-1, CD45, CD11c, CD11b, I-A, CD86, CD80 and CD40, on the culture cells at different culture steps were examined. (The gels have been cropped; for full gel images, see Supplementary Fig. 1) (C) The FCM analysis of the iPS cells. The expressions of Nanog, OCT 3/4, CD309, CD34, c-kit, sca-1, CD45, CD11c and CD11b were analyzed on day 7 of step 1 and day 3 of step 2. The single staining was used for detection each molecule. All experiment data were representative of more three independent experiments and expressed as the mean ± standard error of the mean (SEM) for each experiment except for the levels of c-kit, Sca-1 and CD11b/c in Step2, which reflect the average values from two independent experiments.

**Figure 2:**
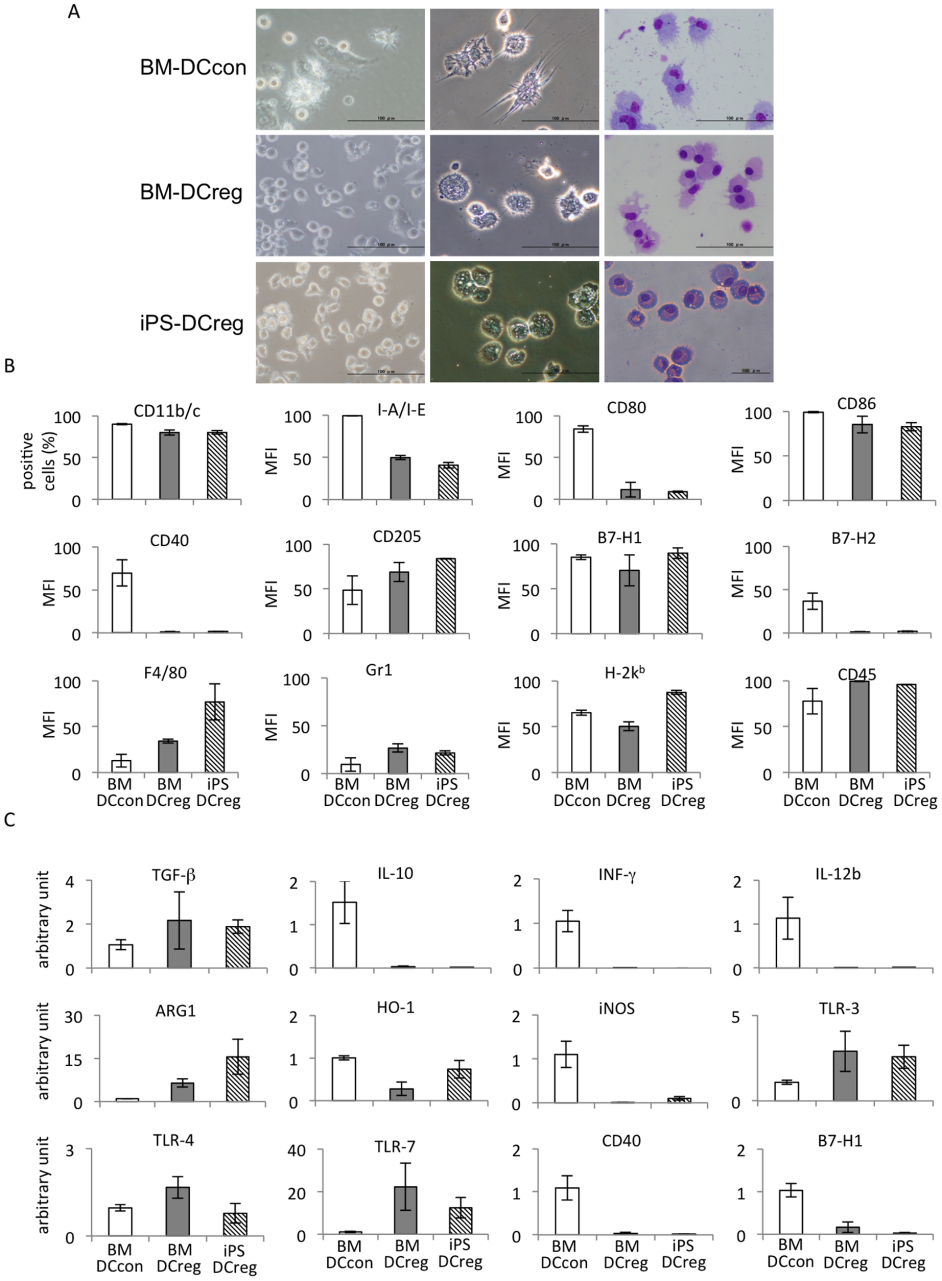
*Characteristics of iPS-DCregs.* (A) The morphology of the BM-DCcons, BM-DCregs and iPS-DCregs under an inverted microscope in the culture dishes (left column) and after the cytospin procedure (middle) and staining with May-Grunwald and Giemsa (right) (*Scale bars* represent 100 μm). (B) The surface molecules, including CD11c, CD11b, IA/IE, CD80, CD86, CD40, CD205, B7-H1, B7-H2, F4/80, Gr-1, H2-K^b^ and CD45, on the BM-DCcons, BM-DCregs and iPS-DCregs. The all of the staining is performed with CD11c (C) The mRNA expressions of TGF−β, IL-10, IFN-γ, IL-12b, ARG-1, HO-1, iNOS, TLR-3, TLR-4, TLR-7, CD40 and B7-H1 on the BM-DCcons, BM-DCregs and iPS-DCregs. All experiment data were representative of more three independent experiments and expressed as the mean ± SEM for each experiment except for the MFI of CD205, B7-H1/2, F4/80, Gr-1 H-2K^b^ and CD45, which reflect the average values from two independent experiments.

The present study investigated iPS-MEF-Ng-38C-2, a previously established murine iPS cell clone, for its capacity to differentiate into functional DCregs. The procedure to induce the differentiation of iPS cells into DCregs was composed of three steps, as shown in Figure 1A. iPS cells were maintained on the feeder layers of PEF. They were similar to ES cells in morphology (Fig. 1A) and growth properties. To initiate the differentiation, the iPS cells were transferred onto OP9 feeder layers. After three days, mesodermally differentiated flat colonies appeared. On day 7, most of the colonies exhibited a differentiated morphology (Fig. 1A). On day 7 of step 1, the cells were harvested using trypsin/EDTA and dissociated into single cells. Subsequently, the cells containing both iPS-derived differentiated cells and OP9 cells recovered from one dish of the step 1 culture were divided into three dishes and cultured in the presence of GM-CSF to start step 2. The next day, homogenous small cells, resembling primitive hematopoietic progenitor cells that expressed CD309, CD34, c-kit and Sca-1 appeared (Fig. 1B, C). The iPS cell-derived differentiated round cells gradually increased and became morphologically heterologous. The addition of exogenous GM-CSF the following day was essential for inducing the propagation of the cells, thus indicating that the cells proliferated in response to GM-CSF. On day 3 of the step 2 culture, the floating cells began to express CD11b (Fig. 1B), thus suggesting their commitment to the myeloid cell lineage. The step 2 culture was continued for 2 ~ 3 days. At the end of step 2, floating or loosely adherent cells were recovered by pipetting and transferred onto bacteriological Petri dishes without feeder cells (step 3) and cultured in the presence of GM-CSF. The next day, the cells were transferred into 24-well hydroplates and cultured in the presence of GM-CSF, TGF-β and IL-10. After five days, most of the floating cells exhibited an irregular shape with some areas of protrusion. The floating cells with areas of protrusion were iPS-DCregs (Fig. 2A). The iPS-DCregs expressed both CD11b and CD11c and began to express CD80, CD86, CD40 and MHC class II on day 1 of the step 3 culture (Figs. 1B, 2B). On day 5 of step 3, the cells were treated with LPS to induce maturation. On day 7 of step 3, the cells were recovered and analyzed. A total of 1 × 10^5^ of iPS cells were obtained after the step 1 and 2 cultures, giving rise to 5.5 × 10^6^ ~ 6.5 × 10^6^ floating cells that could be harvested at the end of step 2. Subsequently, after the step 3 culture, 9 × 10^6^ ~ 1.6 × 10^7^ iPS-DCregs were recovered. In other words, the number of cells increased approximately 90–160 times from the initiation of differentiation to the final differentiation into iPS-DCregs. During the culture process, we observed that the gene features of the iPS cells and the expressions of Nanog and Oct3/4 were downregulated, disappearing by the end of culture step 3 (Fig. 1B).

### Characteristics of the generated iPS-DCregs

We found that the morphology of the iPS-DCregs was similar to that of the BM-DCregs, and compared with the control BM-DCcons, these cells were slightly smaller and rounder (Fig. 2A). Regarding the surface molecular expression, as shown in Figure 2B, we found that the percentage of the expression of CD11b and CD11c double-positive cells was 80–90% in all collected cells, both in the DCs derived from the BM and in the DCregs derived from the iPS cells. These results showed that the purity of the DCregs generated from the iPS cells was sufficient. We also detected other molecules using flow cytometer (FCM) and compared the findings with those of the BM-DCcons as a control. The expressions of secondary co-stimulatory molecules, including CD40, CD80 and B7H2, were decreased in both of the DCregs, while the expressions of CD86 and B7H1 were comparable among three groups of DCs. The expression of the MHC class I molecule H-2K^b^ on iPS-DCregs was slightly higher than both of BM-DCs, while that of the class II molecule IA/IE was lower. In addition, the expressions of other surface molecules, such as CD45, were similar among all groups of DCs, while those of CD205, F4/80 and Gr-1 were higher in the iPS-DCregs than BM-DCcons. Using a quantitative real-time RT-PCR assay, we confirmed that the mRNA expression of CD40 in the iPS-DCregs exhibited the same tendency as that observed in the FCM results, i.e., it was much lower than that observed in either the BM-DCcons or, in some case, BM-DCregs. However, the mRNA expression of B7-H1 differed from the FCM results; it was lower than that observed in the BM-DCcons (Fig. 2C). Furthermore, we also detected the mRNA expressions of cytokines, proteins and toll-like receptors (TLRs) produced by the iPS-DCregs, BM-DCcons and BM-DCregs. As shown in Figure 2C, the mRNA expression of TGF-β in the iPS-DCregs was similar to those observed in the BM-DCregs and higher than that observed in the BM-DCcons, whereas the mRNA expressions of IL-10, IL-12 and interferon (IFN)-γ in the iPS-DCregs were similar to those observed in the BM-DCregs and much lower than those observed in the BM-DCcons. The mRNA expression of Arg-1 in the BM-DCregs was higher than that observed in BM-DCcons, and that observed in iPS-DCregs was much higher than BM-DCregs, while the mRNA expression of HO-1 in the iPS-DCregs was higher than that observed in the BM-DCregs and lower than that observed in the BM-DCcons and the mRNA expression of iNOS in the iPS-DCregs was similar to that observed in the BM-DCregs and much lower than that observed in the BM-DCcons. The mRNA expression of TLR 3 in the iPS-DCregs was similar to those observed in the BM-DCregs and higher than that observed in the BM- DCcons, while the mRNA expression of TLR4 in the iPS-DCregs was a slightly lower than that observed in either the BM-DCregs or BM-DCcons and the mRNA expression of TLR7 in the iPS-DCregs was similar to that observed in the BM-DCregs and much higher than that observed in the BM-DCcons.

### In vitro function of the antigen uptake in the generated iPS-DCregs

**Figure 3:**
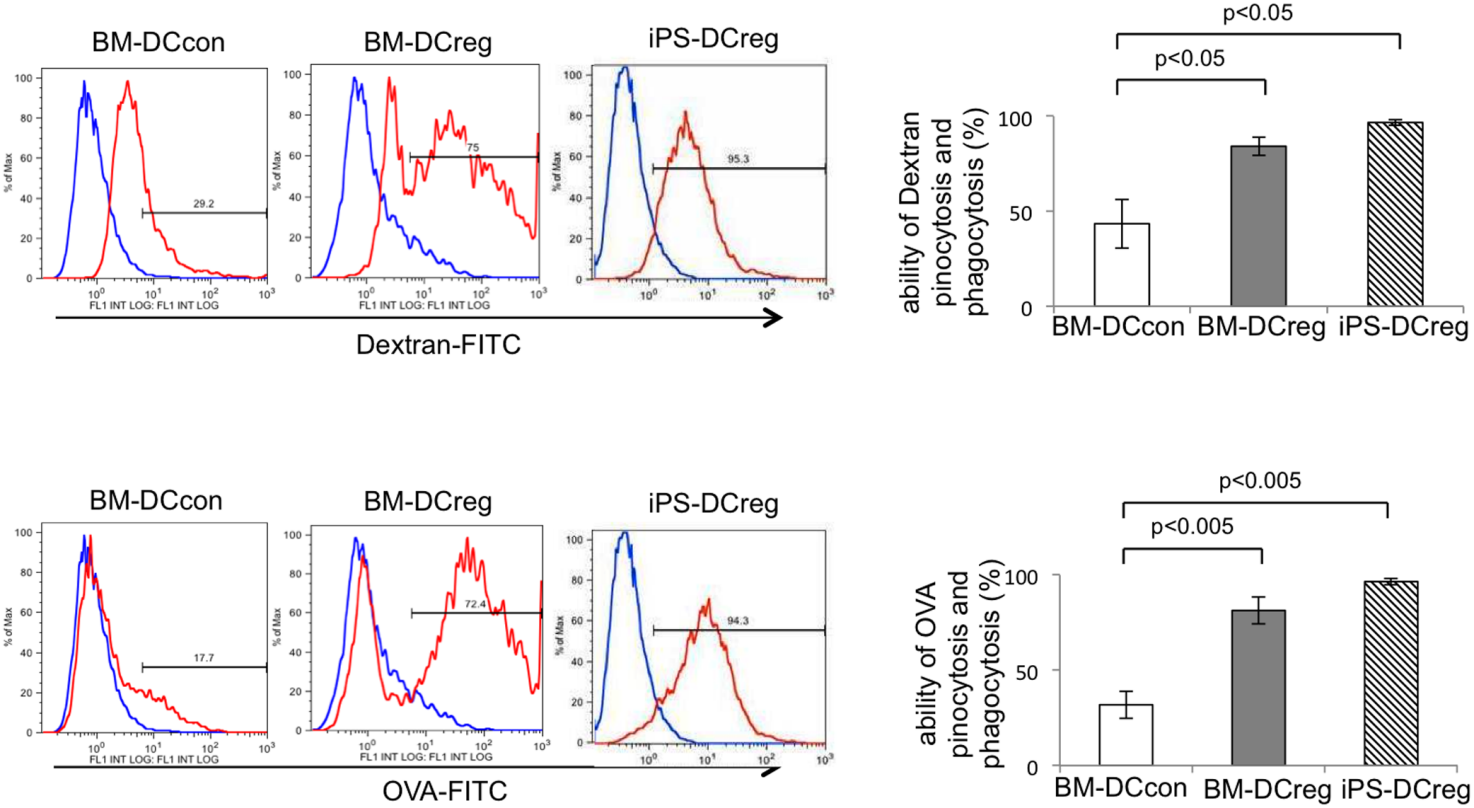
*Antigen uptake analysis of iPS-DCregs.* A carbohydrate antigen (Dextran-FITC) and protein antigen (OVA-FITC) uptake analysis of BM-DCcons, BM-DCregs and iPS-DCregs was performed. The data are representative of more than three independent experiments expressed as the mean ± SEM for each experiment.

In order to examine the endocytic ability in the generated DCs, as shown in Figure 3, we established two antigen uptake assays, using FITC-Dextran and FITC-OVA. We found that the capability for antigen uptake in the iPS-DCregs was comparable to that observed in the BM-DCregs and much higher than that observed in the BM-DCcon. The level of uptake of FITC-Dextran in the iPS-DCregs, BM-DCregs and BM-DCcons was 97%, 84% and 43%, respectively; the difference between the iPS-DCregs and BM-DCcons was significant. The level of uptake of FITC-OVA in these cells was 97%, 81% and 32%, respectively, and the difference between the iPS-DCregs and BM-DCcons was also significant.

### In vitro function of allo-response regulation in the generated iPS-DCregs

**Figure 4:**
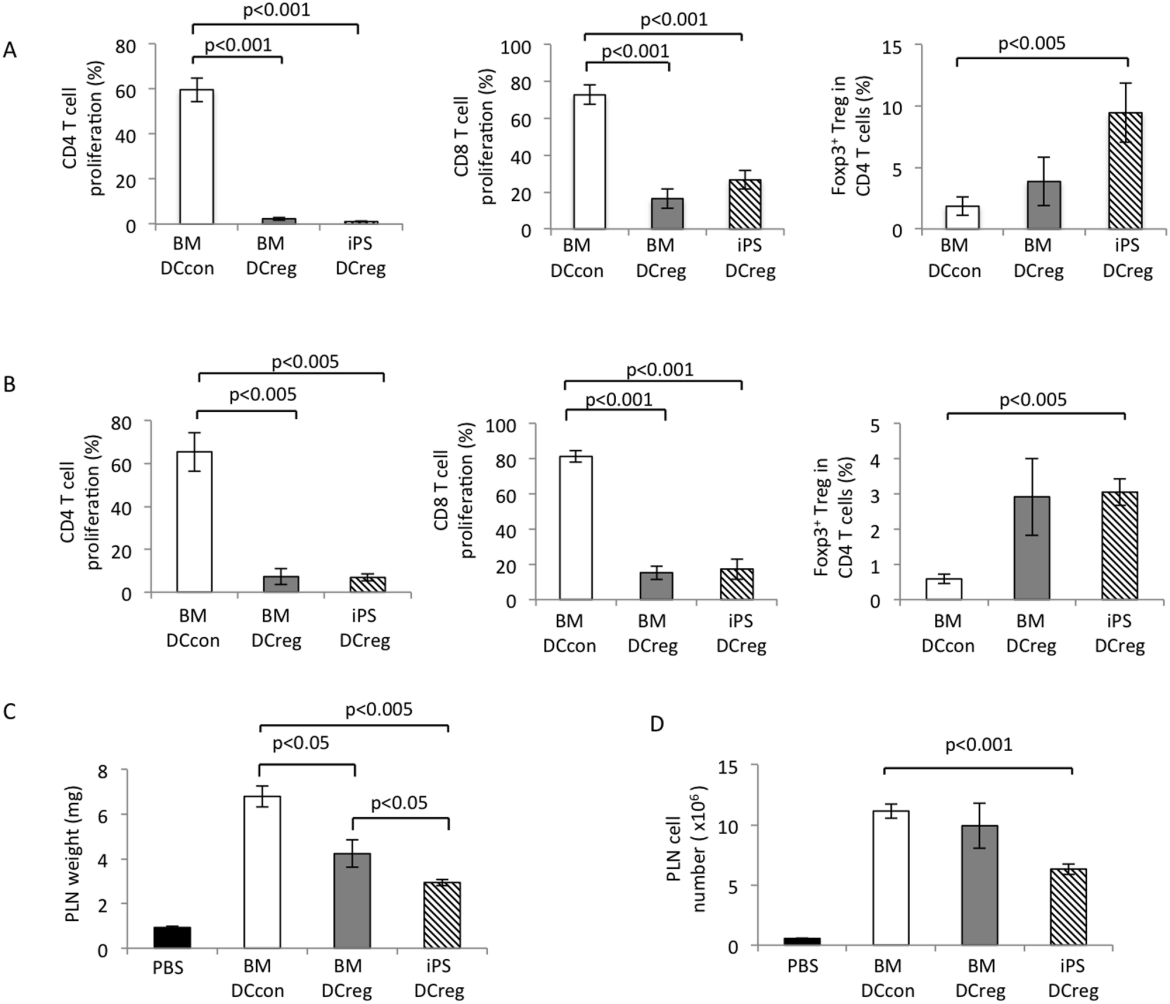
*Allo-response regulation in iPS-DCregs.* (A) The degree of allo-CD4, CD8 T cell proliferation and proportion of the Foxp3-positive Treg cells among the CD4^+^ T cells, stimulated by the BM-DCcons, BM-DCregs and iPS-DCregs was analyzed. The 2 × 10^4^ cells of iPS-DCregs, BM-DCregs and BM-DCcons were used as stimulator and co-cultured with responder T cells (2 × 10^5^), describing as first experiment in the section of MLR in Materials and Methods. (B) The allo-CD4 and CD8 T cell proliferation stimulated by the mature BM-DCcons was regulated by BM-DCregs and iPS-DCregs, not by BM-DCcons. The proportion of Foxp3-positive cells among CD4 T cells was assessed under conditions stimulated by BM-DCcons and regulated by DCs. The X-irradiated BM-DCcons (1 × 10^4^) were used as stimulator and co-cultured with responder T cells (2 × 10^5^) and the iPS-DCregs, BM-DCregs or BM-DCcons (2 × 10^4^), describing as other experiment in the section of MLR in Materials and Methods. (C) The weights (upper) and cell counts (bottom) of the popliteal lymph nodes obtained from the mice injected with BM-DCcons (1 × 10^6^) only or in combination with 5-×10^5^ of BM-DCcons, BM-DCregs or iPS-DCregs into the footpads. The data are expressed as the mean ± SEM from more than three independent experiments.

In order to investigate the effects of iPS-DCregs on allo-reactive T cell responses, we established an Mixed lymphocyte reaction (MLR) assay. As shown in Figure 4A, the mature BM-DCcons efficiently stimulated allo-reactive CD4 and CD8 T cell proliferation. However, the iPS-DCregs showed similar effects to the BM-DCregs, which demonstrated almost no stimulatory effects of allo-reactive CD4 and CD8 T cell proliferation. By contrast, we found that the percentage of Foxp3-positive cells was increased in the CD4 T cells in the iPS-DCreg stimulation group (Fig. 4A, right). This effect was slightly higher than that observed in the BM-DCregs and significantly higher than that observed in the BM-DCcons.

Furthermore, in order to investigate the regulatory/suppressive effects of iPS-DCreg on T cell proliferation, we performed another MLR experiment. As shown in Figure 4B, we found that the iPS-DCregs strongly suppressed the proliferation of both allo-reactive CD4 and CD8 T cells stimulated by mature BM-DCcons. The BM-DCregs also exhibited strong suppression and the suppressive effect of the iPS-DCregs was comparable to the BM-DCregs. When the regulator was replaced with stimulator BM-DCcons, the proliferation of neither CD4 nor CD8 T cells was suppressed. In addition, along with their inhibitory effects on T cell proliferation, the iPS-DCregs increased the proportion of Foxp3-positive cells among CD4 T cells (Fig. 4B, right). The foxp3-positive cell generation of the BM-DCregs was similar to that of the iPS-DCregs. In contrast, when the regulators were replaced with BM-DCcons, the proportion of Foxp3-positive cells in CD4 T cells did not increase.

### In vivo function of allo-response regulation in the generated iPS-DCregs

In order to estimate the suppressive effects of the iPS-DCregs *in vivo*, B6D2F1 mice were immunized subcutaneously with 1 × 10^6^ BM-DCcons in their footpads along with iPS- DCregs. As shown in Figure 4C, the popliteal lymph node (PLN) weight of the mice treated with PBS without BM-DCcons was 0.93 mg. In contrast, the mice immunized with BM-DCcons and treated with BM-DCcons (control) exhibited a lymph node weight of.6.8 mg. Meanwhile, the mice treated with BM-DCregs and BM-DCcons exhibited a lymph node weight of 4.2 mg. In addition, the mice treated with iPS-DCregs and BM-DCcons exhibited a lymph node weight of 2.9 mg. The iPS-DCregs and BM-DCregs significantly reduced the weight of the lymph nodes, with the iPS-DCregs possessing significantly stronger regulatory properties than the BM-DCregs.

Moreover, as shown in Figure 4D, the number of lymphocytes was increased in the mice injected with BM-DCcons. When the mice were treated with PBS, the number of popliteal lymphocytes was 0.56 × 10^6^. Furthermore, the mice treated with BM-DCcons (control) and BM-DCcons had 11 × 10^6^ popliteal lymphocytes. Meanwhile, the mice treated with BM-DCregs exhibited a lymphocyte count of 9.9 × 10^6^, while the mice treated with iPS-DCregs demonstrated a lymphocyte count of 6.3 × 10^6^. Taken together, these data show that iPS-DCregs effectively inhibit allo-immune lymphocyte proliferation *in vivo*. Moreover, even when the number of DCregs was increased by two or four times, the inhibitory effects were not obviously synergistic,suggesting that half the number of Dcreg relative to stimulator cells was sufficient to suppress lymphocyte proliferation.

## Discussion

In the present study, we demonstrated a method of generating DCregs from iPS cells and evaluated the characteristics of the produced cells. We used OP9 stromal cells as feeder cells accompanied by the combination of exogenous GM-CSF and the anti-inflammatory cytokines IL-10 and TGF-β as additive components to generate DCregs. We found that our present generation method successfully produced DCs that exhibited low expressions of MHC and co-stimulatory molecules and demonstrated immune regulatory functions both *in vitro* and *in vivo*. We considered that our culture procedure is suitable for generating efficient regulatory DCs from iPS cells.

iPS cells are similar to ES cells, which are characterized by pluripotency and an infinite capacity for propagation. iPS cells can be differentiated into various cells belonging to the three germ layers, as demonstrated in analyses of teratomas generated from murine and human iPS cells^22^,^26^. Nanog and Oct3/4 are the iconic nuclear transcription factors of iPS cells, which also exhibit retention of pluripotency. During our process of generating iPS-DCregs, the mRNA expression of the iPS cells revealed that the expressions of Nanog and Oct3/4 gradually decreased in the step 2 differentiated cells within days and eventually disappeared by step 3 (Fig. 1B, C). These data demonstrate that the final recovered culture cells were completely differentiated and that the cells can be used safely as stable effector cells for the treatment of various immune-related diseases, including allograft rejection and autoimmune disease.

DCs, like all other leukocytes, are developed from BM-derived hematopoietic stem cells (HSCs)^27^. Therefore, when attempting to generate DCregs from iPS cells, the first thing to consider is how to induce the differentiation of iPS cells toward to HSCs. OP9 is a bone marrow stromal cell line originated from macrophage colony-stimulating factor (M-CSF)-defective op/op mice^28^. The generation of various hematopoietic cells from ES cells using OP9 cells as feeder cells has been reported, including granulocytes, erythrocytes, B lymphocytes and osteoclasts^29^,^30^,^31^. Senju et al. demonstrated that OP9 cells are also suitable for generating ES/iPS-DCs as culture feeder cells^25^,^32^. According to the above evidence, we selected OP9 cells as culture feeder cells in the present study. CD309, CD34, c-kit and sca-1 are considered to be the primary markers of HSCs and are often used to separate HSCs from bone marrow stem cells^33^. Along with the maturation of cells, these molecules are gradually weakened until they disappear. In our generation process, we found that CD309, CD34, c-kit and sca-1 appeared at the end of step 1 in the differentiated cells and were maintained in the step 2 and 3 differentiated cells until they finally disappeared on day 7 of step 3, the end of the entire generation process (Fig. 1B, C). These phenomena suggest that our iPS-DCreg generation method is suitable for inducing HSCs from iPS cells.

From step 2, which involved monocyte and dendritic cell progenitor (MDP) differentiation from HSC-like cells^27^, we added the cytokine GM-CSF to the culture. In GM-CSF-supplemented marrow cultures, DCs arise from cellular aggregates that are attached to the marrow stroma^34^. The aggregates become covered with sheet-like cell processes and eventually release typical single dendritic cells. During the step 2 process, we observed a phenomenon in the iPS cells similar to that observed in the bone marrow cell culture, i.e., the DC progenitor-like cellular aggregates gradually arose from the cells mixed with OP9 cells, accompanied by the increased expressions of CD11b and CD11c in these floating cells. These results suggest that GM-CSF is also suitable for stimulating iPS cells to generate DCs.

In order to generate DCregs, we added the cytokines IL-10 and TGF-β to the step 3 culture. TGF-β and IL-10 are anti-inflammatory cytokines that can prevent DC maturation and/or functioning both *in vitro* and *in vivo*^12^,^14^. Sato et al. reported the use of successfully generated DCregs obtained from culturing BM cells with GM-CSF, IL-10 and TGF-β as therapeutic agents in mice^35^. The authors also reported that human modified DCs obtained from culturing monocytes with GM-CSF, IL-4, IL-10 and TGF-β also act as DCregs, inducing potent immunoregulation *in vitro*^9^.

The primary characteristics of DCregs include a weak expression of MHC and co-stimulatory molecules and a weak ability to activate effector T cells, while inducing the proliferation of regulatory T cells and the anergy of autoreactive T cells in order to stimulate immune tolerance^2^,^3^,^17^. In addition, studies have demonstrated that IL-10 inhibits the stimulatory capacity of APCs and that the inhibitory effect of IL-10 is due to the downregulation of MHC class II molecules and the co-stimulatory molecules B7-H1/2 and intercellular adhesion molecule-1^36^,^37^,^38^. These effects may also contribute to developing iPS-DCregs, since the iPS-DCregs produced using the present method exhibited much lower expressions of CD40, CD80 and MHC class II, whereas the expression of CD86 was comparable to that observed in the BM-DCcons and similar results were observed in the BM-DCregs (Fig. 2B). These results are somewhat different from those of Sato's reports, which showed that all of the expressions of CD40, CD80, CD86 and MHC class II were reduced in the BM-DCregs, no matter which strain of mice were used^35^. An analysis of the functions of the generated DCs found that the mature BM-DCcons efficiently stimulated allogeneic CD4 and CD8 T cell proliferation as APCs, whereas the iPS-DCregs and BM-DCregs exhibited almost no stimulatory capability with respect to allogeneic CD4 T cell proliferation and only weakly stimulated allogeneic CD8 T cell proliferation (Fig. 4A).

In addition, we found that the iPS-DCregs, similar to the BM-DCregs, exhibited significantly higher antigen uptake efficiency than the LPS-stimulated BM-DCcons, which were considered to be mature DCcons (Fig. 3), consistent with the findings of previous reports^3^. We also found that the degree of antigen uptake and allo-reactive T cell stimulation induced by the LPS-stimulated iPS-DCregs and BM-DCregs was comparable to that of the DCregs not stimulated with LPS (data not shown). These results indicate that iPS-DCregs possess the stable properties of immature DCs, regardless of whether they are stimulated with LPS.

As a representative effect of the DCregs, the suppression of allo-reactive T cell proliferation and the generation of Treg cells were observed in the iPS-DCregs. We found that the iPS-DCregs strongly suppressed the proliferation of both allo-CD4 and CD8 T cell proliferation stimulated by mature BM-DCcons. Moreover, in the *in vivo* PLN assay experiment, we confirmed that the iPS-DCregs downregulated the allo-immune response. Furthermore, we found that the proportion of Foxp3-positive cells in CD4 T cells was increased by co-culture with the generated iPS-DCregs in both types of MLR studies. The transcription factor Foxp3 was identified to be the main factor associated with Treg cells, and several studies have reported that Foxp3 plays an important role in the development and function of naturally occurring CD4 Treg cells^39^,^40^. Treg cells have been proven to play key roles in the maintenance of immunologic self-tolerance and to act as negative controls in a variety of physiological and pathological immune responses^41^,^42^. Therefore, iPS-DCregs have the potential capacity to generate Treg cells that contribute to the suppression of allo-reactive T cell proliferation. The further characterization of the iPS-DC, for instance, the stability of DC phenotype and function, is necessary for the clinical application.

In conclusion, we herein reported an effective method for generating functional DCregs from iPS cells. Further studies are required to clarify the exact mechanisms of how iPS-DCregs regulate allo-immune reactions and whether iPS-DCregs can be used to treat allograft rejection and/or autoimmune diseases.

## Methods

### Cell lines, cytokines, chemicals and peptides

The murine embryonic fibroblast-derived iPS cell line (iPS-MEF-Ng-38C-2, H-2k^b/d^), a gift from Dr. S. Yamanaka, Kyoto University, was maintained in Dulbecco's modified Eagle's medium (DMEM) containing 20% ES cell-qualified fetal calf serum (FCS; GIBCO-Invitrogen, Carlsbad, CA), 1,000 U/ml of leukemia inhibitory factor, 50 U/ml of penicillin, 50 mg/ml of streptomycin, nonessential amino acids and 50 μM of 2-mercaptoethanol (2-ME) on feeder cell layers of mitomycin C-treated murine primary embryonic fibroblasts (PEFs). Murine bone marrow stromal cells, OP9, were maintained in DMEM supplemented with 20% FCS and seeded onto gelatin-coated dishes before use as feeder cells. Recombinant murine IL-4, IL-10, TGF-β and GM-CSF were purchased from Peprotec (London, U.K.). Lipopolysaccharide (LPS) derived from Escherichia coli was purchased from Sigma Chemical (St. Louis, MO).

### Mice

Female Foxp3/GFP knock-in C57BL/6 (H-2k^b^), B6D2F1 (C57BL/6 × DBA/2, H-2k^b/d^) mice 8 to 12 weeks of age weighing 20 ~ 25 g were purchased from Jackson Laboratory (Bar Harbor, ME) and Shizuoka Laboratory Animal Center (Shizuoka, Japan). All mice were maintained under standard conditions and fed rodent food and water. All animal experiments were approved by the Committee on the Care and Use of Laboratory Animals at the National Research Institute for Child Health and Development, and performed according to their recommendation.

### Differentiation culture

The procedure for inducing the differentiation of iPS cells into DCs is composed of three steps modified according to the method established by Senju et al.^25^ (Fig. 1A). Step 1: The iPS cells were suspended in α-MEM supplemented with 20% FCS and seeded (1 × 10^5^ cells per dish) onto OP9 cell layers in 100-mm dishes. On day 7, the cells were treated with PBS with 0.25% trypsin/1 mM EDTA for 10 minutes, recovered with medium containing FCS and subjected to step 2 of the culture or stocked frozen for future use. Step 2: Cells harvested from the step 1 culture were suspended in α-MEM supplemented with 20% FCS, GM-CSF (10 ng/ml) and 2-ME (50 μM) and plated in 100-mm dishes. The cells containing both iPS-derived cells and OP9 cells recovered from one dish of the step 1 culture were divided into three dishes. The next day, the same culture medium with GM-CSF and 2-ME was added. Thereafter, 2–3 days after the passage, floating cells were recovered by pipetting and subjected to the step 3 culture or stocked frozen. Step 3: The cells were transferred to 2 × 10^6^ cells/100-mm bacterial Petri dishes (Locus; Tokyo, Japan) without feeder cells and cultured in RPMI-1640 medium supplemented with 10% FCS, GM-CSF and 2-ME. On day 2 after beginning step 3, the cells were transferred to 24-well hydroplates (5 × 10^5^ per well, CellSeed Inc., Tokyo, Japan) cultured in RPMI-1640/10% FCS supplemented with GM-CSF (20 ng/ml), IL-10 (20 ng/ml), TGF-β (20 ng/ml) and 2-ME. On day 5 or 6 after beginning step 3, the cells were stimulated with LPS (1 μg/ml) for and additional two days then collected for the analysis.

### Generation of conventional DCs and regulatory DCs from BM cells

Femoral and tibial BM cells were obtained from female B6D2F1 mice. To generate conventional DCs (BM-DCcons), the cells were cultured in RPMI-1640 medium supplemented with 10% FCS, 10 ng/ml of GM-CSF, 10 ng/ml of IL-4 and 50 μM of 2-ME for five days in 24-well cell culture plates (Greiner bio-one Japan, Tokyo, Japan). For maturation, 1 μg/ml of LPS was added and, after an additional two days of culture, the cells were collected for the analysis. To generate regulatory DCs (BM-DCregs), the cells were cultured in RPMI-1640 medium supplemented with 10% FCS, 20 ng/ml of GM-CSF, 20 ng/ml of IL-10, 20 ng/ml of TGF-β and 50 μM of 2-ME for seven days in 24-well cell culture plates.

### Microscopic analysis

A bright-field microscopic analysis was performed using an inverted microscope (IX70, Olympus, Tokyo, Japan). The cytospin technique was carried out using the Shandon Cytospin®4 Cytocentrifuge (Thermo Fisher Scientific Inc., Waltham, MA). The cytospin specimens were stained with May-Grunwald-Giemsa (Merck; Darmstadt, Germany). Microscopic images were captured using a digital camera unit DP70 (Olympus).

### Flow cytometric analysis

The test cells were collected and suspended in PBS and then incubated at 4°C for 20 minutes with optimal concentrations of various fluorescence monoclonal antibodies. The following monoclonal antibodies (mAb) conjugated with fluorescence isothiocyanate (FITC), PE, Alexa Fluor 647 or APC were used for staining: anti-mouse Nanog (clone eBioMLC-51, rat IgG2a, eBioscience, San Diego, CA), anti-mouse OCT3/4 (clone EM92, rat IgG2a, eBioscience), anti-mouse CD309 (clone Avas 12, rat IgG2a, eBioscience), anti-mouse CD34 (clone MEC14.7, rat IgG2a, eBioscience), anti-mouse CD117 (c-kit) (clone 2B8, rat IgG2a, Biolegend, San Diego, CA), anti-mouse Ly6A/E (Sca-1) (clone D7, rat IgG2a, Biolegend) and anti-mouse CD45 (clone 30-F11, rat IgG2b, Biolegend). For Nanog and OCT3/4, the eBioscience Foxp3 Staining buffer set (eBioscience) was used. The following mAbs conjugated with FITC, PE, APC, PE-Cy5.5, PE-Cy7 or APC-Cy7 were used for staining: anti-mouse CD11b (clone M1/70, rat IgG2b, Biolegend), anti-mouse CD11c (clone N148, hamster IgG, Biolegend), anti-mouse CD40 (clone 3/23, rat IgG2a, Biolegend), anti-mouse CD80 (clone 16-10A1, hamster IgG, Biolegend), anti-mouse CD86 (clone GL-1, rat IgG2a, Biolegend) and anti-mouse IA/IE (clone M5/114.15.2, rat IgG2b, Biolegend). For the T cell analysis, the following mAbs conjugated with FITC, PE, APC or PE-Cy7 were used for staining: anti-mouse CD4 (clone RM4-5, rat IgG2a, Biolegend), anti-mouse CD8a (clone 53-6.7, rat IgG2a, Biolegend), anti-mouse CD25 (clone PC61, rat IgG2a, Biolegend), rat IgG2a control (clone RTK2758, Biolegend), rat IgG2b control (clone RTK 4530, Biolegend) and hamster IgG control HTK888). The stained cells were analyzed using flow cytometer (FCM) (Gallios®, Beckman Coulter, Brea, CA or Attune®, Life technologies-ABI, Carlsbad, CA), and the data were analyzed using the FlowJo software program (Tree Star, Ashland, OR).

### RNA preparation and quantitative reverse transcriptase-polymerase chain reaction (qRT-PCR)

Total RNA was extracted from the frozen renal tissue samples using ISOGEN (NipponGene, Tokyo, Japan). Each 0.8-μg aliquot of RNA was reverse-transcribed to cDNA using oligo (dT) primers and Super Script™ reverse transcriptase (Life technologies-Invitrogen). Primers amplifying the murine mRNA regions and a specific Taqman probe were designed using the Primer Express software package (Applied Biosystems). Quantitative RT-PCR was performed using the TaqMan system on the Applied Biosystems PRISM7900 instrument (Life technologies-ABI), and the experiments were conducted using 0.9 mM of each primer in a final reaction volume of 25 μl of Premix Ex Taq™ (Takara Bio Inc., Shiga, Japan). The PCR cycling conditions were as follows: 50°C for two minutes, 95°C for 15 minutes and 50 cycles of 95°C for 30 seconds, 60°C for one minute and 25°C for two minutes. The normalized Ct value of each gene was obtained by subtracting the Ct value of 18s rRNA.

### Antigen uptake assay

iPS-DCs were seeded into 24-well cell culture plates (1 × 10^6^ cells per well) cultured in 10% FCS RPMI-1640. For antigen uptake, a portion of the cells were incubated with 20 μg/ml fluorescein ovalbumin (O23020, Life technologies-Molecular Probes) in 37°C or 4°C for three hours, while another portion of the cells were incubated with 1 mg/ml FITC-dextran (D-1845, Life technologies-Molecular Probes) at 37°C and 4°C for 30 minutes, respectively. After incubating, the cells were collected and washed with PBS, then stained with PE-Cy7-CD11b and APC-CD11c. The stained cells were analyzed using FCM.

### Mixed lymphocyte reaction (MLR)

Splenic T cells were isolated from female Foxp3/GFP knock-in C57BL/6 mice using a nylon-wool column (Wako, Osaka, Japan) and stained with Violet Proliferation Dye 450 (Becton Dickinson) then used as responders. Two types of MLR experiments were performed. In the first experiment, the generated iPS-DCregs, BM-DCregs and BM-DCcons were used as stimulator cells, and all cells were studied at three amounts (1 × 10^4^, 2 × 10^4^ and 4 × 10^4^) and co-cultured with responder T cells (2 × 10^5^) in the wells of 96-well round-bottomed culture plates (Greiner bio-one) for four days.

In the other experiment, matured BM-DCcons were used as stimulator cells (1 × 10^4^) and treated with X-ray irradiation (20 Gy) and co-cultured with responder T cells (2 × 10^5^) and the regulators (iPS-DCregs, BM-DCregs or BM-DCcons), and all cells were studied at three amounts (1 × 10^4^, 2 × 10^4^ and 4 × 10^4^) in the wells of 96-well round-bottomed culture plates (Greiner bio-one) for four days. At the end of the culture, the cells were harvested and stained with PE-Cy7-CD8 and APC-CD4. The stained cells were analyzed using FCM.

### In vivo lymphocyte proliferation assay

BM-DCcons were injected into the footpads of the hind feet of female Foxp3/GFP knock-in C57BL/6 mice at 1 × 10^6^ cells/foot. The mice were injected with iPS-DCregs or BM-DCregs or BM-DCcons at 5 × 10^5^, 1 × 10^6^ or 2 × 10^6^ cells/foot accompanied by BM-DCcons. The control mice were injected with the same amount of PBS. After seven days of injection, the popliteal lymph nodes were harvested and weighed. The lymphocytes were separated and analyzed.

### Statistical analysis

Student's *t*-test was used to compare the paired and unpaired analyses. *P* values of less than 0.05 were considered to be statistically significant.

## Author Contributions

Q.Z., S.I., Y.K. and H.H. performed experiment. M.F. and X.-K.L. wrote the manuscript. M.F., S.C., J.X. and X.-K.L. designed experiment and analyzed data.

## Supplementary Material

Supplementary Information
