# Supplementary material for: Generation and characterization of regulatory dendritic cells derived from murine induced pluripotent stem cells
Source: Sci Rep. 2014 Feb 5;4:3979. doi: 10.1038/srep03979 (PMC3913921; doi:10.1038/srep03979)
Supplement: Supplementary Information [file srep03979-s1.pdf]

Title:

Generation and characterization of regulatory dendritic cells derived from murine induced pluripotent stem cells

Authors:

Qi Zhang[1, 2], Masayuki Fujino[2, 3], Shizue Iwasaki[2], Hiroshi Hirano[2], Songjie Cai[2], Yuya Kitajima[2], Jinhua Xu[1], Xiao-Kang Li[1, 2]

Affiliations:

[1]Department of Dermatology, Huashan hospital, Fudan University, Shanghai, China;

[2]Division of Transplantation Immunology, National Research Institute for Child Health and Development, Tokyo, Japan;

[3]AIDS Research Center, National Institute of Infectious Diseases, Tokyo, Japan.

[*]Correspondence:

Xiao-Kang Li, M.D., Ph.D., Division of Transplantation Immunology, National Research Institute for Child Health and Development, 2-10-1 Okura, Setagaya-ku, Tokyo, 157-8535 Japan.

Tel: +81-3-3416-0181; Fax: +81-3-3417-2864; E-mail: ri-k@ncchd.go.jp

&

Jinhua Xu, M.D., Ph.D., Department of Dermatology, Huashan Hospital, Fudan University, 12 Wulumuqi Middle Road, Shanghai, 200040 China.

Tel:+86-21-5288-9999; Fax:+86-21-6248-9191; E-mail: xjhhuashan@yahoo.com.cn

Supplementary figure legends

Supplementary Figure 1: Un-cropped gel images of RT-PCR. The mRNA expression of the iPS cells in each step of differentiation into DCregs. The mRNA expressions of major molecules, including (A) Nanog, (B) OCT 3/4, (C) CD34, (D) Sca-1, (E) CD45, (F) CD11c, (G) CD11b, (H) I-A, (I) CD86, (J) CD80, (K) CD40 and (L) GAPDH, on the culture cells at different culture steps were examined.

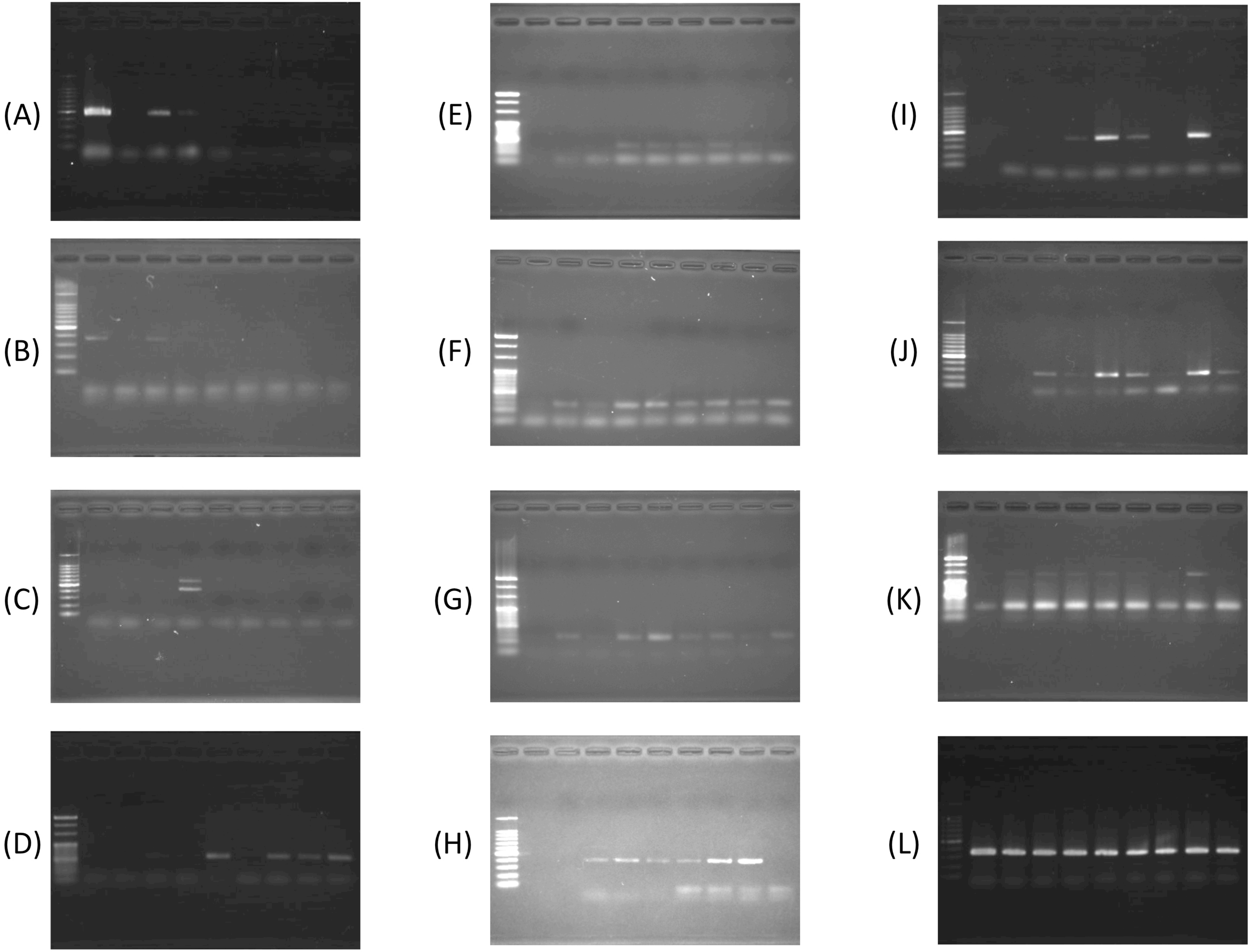
(A)
(B)
(C)
(D)
(E)
(F)
(G)
(H)
(I)
(J)
(K)
(L)
